# Conclusive evidence for hexasomic inheritance in chrysanthemum based on analysis of a 183 k SNP array

**DOI:** 10.1186/s12864-017-4003-0

**Published:** 2017-08-07

**Authors:** Geert van Geest, Roeland E Voorrips, Danny Esselink, Aike Post, Richard GF Visser, Paul Arens

**Affiliations:** 1 Plant Breeding, Wageningen University and Research, P.O. Box 386, 6708 PB Wageningen, the Netherlands; 2 Deliflor Chrysanten B.V, Korte Kruisweg 163, 2676 BS Maasdijk, the Netherlands

**Keywords:** Polyploid, SNP retrieval, Allelic expression bias, SNP array, RNA-seq, Polysomic, Disomic, Hexaploid

## Abstract

**Background:**

Cultivated chrysanthemum is an outcrossing hexaploid (2n = 6× = 54) with a disputed mode of inheritance. In this paper, we present a single nucleotide polymorphism (SNP) selection pipeline that was used to design an Affymetrix Axiom array with 183 k SNPs from RNA sequencing data (1). With this array, we genotyped four bi-parental populations (with sizes of 405, 53, 76 and 37 offspring plants respectively), and a cultivar panel of 63 genotypes. Further, we present a method for dosage scoring in hexaploids from signal intensities of the array based on mixture models (2) and validation of selection steps in the SNP selection pipeline (3). The resulting genotypic data is used to draw conclusions on the mode of inheritance in chrysanthemum (4), and to make an inference on allelic expression bias (5).

**Results:**

With use of the mixture model approach, we successfully called the dosage of 73,936 out of 183,130 SNPs (40.4%) that segregated in any of the bi-parental populations. To investigate the mode of inheritance, we analysed markers that segregated in the large bi-parental population (*n* = 405). Analysis of segregation of duplex x nulliplex SNPs resulted in evidence for genome-wide hexasomic inheritance. This evidence was substantiated by the absence of strong linkage between markers in repulsion, which indicated absence of full disomic inheritance. We present the success rate of SNP discovery out of RNA sequencing data as affected by different selection steps, among which SNP coverage over genotypes and use of different types of sequence read mapping software. Genomic dosage highly correlated with relative allele coverage from the RNA sequencing data, indicating that most alleles are expressed according to their genomic dosage.

**Conclusions:**

The large population, genotyped with a very large number of markers, is a unique framework for extensive genetic analyses in hexaploid chrysanthemum. As starting point, we show conclusive evidence for genome-wide hexasomic inheritance.

**Electronic supplementary material:**

The online version of this article (doi:10.1186/s12864-017-4003-0) contains supplementary material, which is available to authorized users.

## Background

The ability to genotype large numbers of polymorphisms is of major importance for breeding and genetic analysis. Costs for detection and genotyping of a large number of polymorphisms are still decreasing, and therefore become available to an increasing number of agriculturally important plant species, including polyploids. Genetic analysis in polyploids is less straightforward compared to diploids. An example is cultivated chrysanthemum, which is an outcrossing hexaploid (2n = 6× = 54) and has been classified as a segmental allopolyploid [1].

Polymorphism detection in species without a reference genome is restricted to methods using a reduced representation of the genome, like restriction enzyme based selection methods (e.g. RADseq or GBS [2, 3]), bait capture [4, 5] or RNA sequencing (RNA-seq; e.g. [6, 7]). RNA-seq is particularly useful for polymorphism detection for multiple reasons. First, discovered polymorphisms are in genic regions. Therefore, they have a high chance to represent or to be close to polymorphisms causative for an investigated phenotype. Secondly, lower single nucleotide polymorphism (SNP) densities are expected in expressed sequences, which is an advantage in highly heterozygous polyploid species, as polymorphisms in flanking regions interact with marker assays. Thirdly, markers are in regions with transcribed genes, which generally have high recombination rates [8], and discovered markers are therefore particularly useful for linkage mapping. Lastly, RNA-seq gives a representation of the transcriptome that helps building resources useful for other analyses.

A disadvantage of the use of RNA-seq is possible discordance between the expression of an allele and the allele’s dosage in genomic DNA. Expression of certain alleles might even be completely absent. This feature is also referred to as allelic expression bias, and specifically occurs in allopolyploids [9]. In hexaploid wheat for example, most expressed genes present in all subgenomes show expression bias towards one of the subgenomes, and expression is lost in at least one of the subgenomes for most genes [10]. Another challenge for the use of RNA-seq for polymorphism detection is the de novo assembly of raw reads. Multiple splice variants in the transcriptome can represent one gene locus, which makes reconstruction of a locus on the genome challenging. For outcrossing polyploids like chrysanthemum, high heterozygosity [11] is another challenge. Large variation between alleles makes it difficult to distinguish alleles from homologous genes. This will result in false polymorphism calls if gene homologues are assembled together in one contig or in the inability to detect polymorphisms if alleles are assembled into different contigs [12].

High throughput genotyping of SNP polymorphisms using SNP assays is common in diploids [13, 14]. Several applications in the allopolyploids wheat and strawberry and in the tetraploids potato and rose have been published [6, 7, 15, 16]. SNP assays like Illumina Infinium, Affymetrix Axiom and LGC KASP provide signal intensities for each of the two allelic probes based on fluorescence. Allele dosage can be deduced from clusters, which can be visualised by plotting the two allelic signal intensities against each other. For diploid SNPs, including subgenome specific SNPs in polyploids with disomic inheritance, three clusters are expected: two homozygous and one heterozygous. SNPs in polyploids with polysomic inheritance, and SNPs polymorphic in more than one subgenome in disomic polyploids, show at maximum five clusters for tetraploids and seven for hexaploids. The assignment of a dosage to such clusters can be challenging, as clusters are often shifted [17], and resolution of the assay might not suffice [18]. High heterozygosity aggravates these issues, as undetected adjacent polymorphisms can influence a SNP assay.

The mode of inheritance has large implications for genetic analysis in polyploid organisms. In general, the mode of inheritance relates to the origin of ploidy of the organism: whether it is allopolyploid or autopolyploid. Disomic inheritance is usually a feature of allopolyploids and polysomic inheritance of autopolyploids. Ramsey and Schemske [19] defined allopolyploids as polyploid organisms that have originated from interspecific hybridization in which genomes of the progenitors are retained, and autopolyploids as organisms originated from within a single species, often as a result of unreduced gametes. Since these definitions address origin, but not the mode of inheritance, allopolyploids not necessarily have disomic inheritance, and autopolyploids do not necessarily have full polysomic inheritance [20, 21]. An example is cultivated rose, which originated from multiple interspecific crosses [22], which makes it an allopolyploid, but its mode of inheritance is mostly polysomic [23, 24]. Also intermediate modes of inheritance exist in several polyploid organisms [25–28]. As the most widely used definitions of allopolyploidy and autopolyploidy are on the origin, and not on the mode of inheritance, in this paper we only aim to separate disomic from polysomic inheritance.

The mode of inheritance in chrysanthemum has been under discussion [1, 29]. Cultivated chrysanthemum is generally assumed to have originated from multiple species, making it an allopolyploid [30, 31]. However, evidence is scarce. Despite the presumed allopolyploidy, there is evidence for polysomic inheritance in chrysanthemum. Cytological studies of cultivated chrysanthemum report presence of multivalents during meiosis, although most chromosomes pair as bivalents [32, 33]. Multivalents will lead to recombination between all pairing homologous chromosomes and therefore indicate polysomic inheritance. The relatively high number of bivalents is not necessarily an indication of prevalence for disomic inheritance, as bivalents could represent a pairing event between any of the homologous chromosomes [34], and bivalent formation is known to be under genetic control in chrysanthemum [35].

In addition to cytological observations, hexasomic inheritance is also suggested by the analysis of segregation of molecular markers. Two studies showed that alleles from a single multi-allelic SSR marker have independent assortment, which is only possible with hexasomic inheritance [1, 36]. Another strong line of evidence for polysomic inheritance is from the earlier work of Langton [37] on the inheritance of a flower colour trait regulated by a single dominant allele. In the study, a self-compatible simplex (dosage of one) individual is selfed. The duplex (dosage of two) progeny of this selfing is crossed with nulliplex (dosage of zero) genotypes. In the case of disomic inheritance, the two alleles in the duplex parent would be on the same sub-genome in the duplex parent, and therefore should not segregate. However, in the resulting populations, the trait segregates in ratios as expected from hexasomic inheritance. Despite the strong evidence for polysomic inheritance, the observations on SSR markers and flower colour are based on a few loci; other locations on the genome might show disomic inheritance. In order to acquire a genome-wide overview of the mode of inheritance, segregation analysis of a large number of markers distributed over the entire genome is required.

Multi-allelic SSR markers are scarce, and self-compatibility is difficult to obtain in chrysanthemum. However, analysis of segregation of high numbers of SNP markers in large outcrossing F1 populations can also provide evidence for the mode of inheritance. One of such analyses involves segregation of markers that are duplex in one parent and nulliplex in the other. If inheritance is disomic and the duplex alleles are on the same subgenome, all progeny will be simplex (one). Existence of these non-segregating duplex x nulliplex (2 × 0) markers therefore indicates disomic inheritance. If the two alleles are on different subgenomes, disomic inheritance will lead to a 1:2:1 segregation of the dosages 0, 1 and 2. Hexasomic inheritance will lead to 1:3:1 segregation in all cases. Studies that analysed deviations from those types of segregation, in general found duplex markers both fitting hexasomic inheritance as well as disomic inheritance [1, 29, 36, 38]. Particularly in small populations genotyped with dominant markers, these tests are not powerful, because the segregation distributions (3:1 versus 4:1) are close to each other. Testing for segregation of a large number of markers in a large population with co-dominant markers, probably leads to less ambiguous conclusions.

A third method for estimation of the mode of inheritance is analysis of repulsion linkage [39]. Estimates of recombination frequencies (*r*) assuming disomic (diploid-like) inheritance between markers in repulsion that approach zero indicate disomic inheritance. In the case of hexasomic inheritance, pairing should be random with all pairs of homologues chromosomes. In that case, the minimum diploid maximum likelihood estimator of *r* of markers in repulsion should be 0.4 [40]. In chrysanthemum, earlier analysis of repulsion linkage pointed towards hexasomic inheritance [1].

In this paper, we present a SNP selection pipeline for chrysanthemum from RNA-seq data (1), a method for dosage scoring in hexaploids from bi-allelic probe fluorescence (2) and validation of selection steps in the SNP selection pipeline (3). The resulting genotypic data is used to draw conclusions on the mode of inheritance in chrysanthemum (4) and allelic expression bias (5).

## Results

### RNA sequencing, assembly, and alignment

RNA-seq resulted in an average of 100.4 M reads for the deep-sequenced parents of the large population (405 individuals, POP1) and on average 70.4 M reads for the 11 other sequenced cultivars (Additional file 1). Sequence assembly resulted in 270,186 contigs for the female parent and 275,397 contigs for the male parent (Additional file 2). Clustering with uclust [41] at 99% similarity reduced the number of contigs to 227,213 and 231,634 respectively. As the average contig length in the female parent was longer and total number of contigs was lower, the assembly of the female parent was considered as higher quality and therefore used as reference transcriptome. Mapping reads of all cultivars to this assembly using bwa-mem resulted in an average alignment rate of 88.6 ± 0.9%, for bowtie2 this was 81.6 ± 0.7%.

### SNP filtering

In total 183,130 SNPs were included in the array. Of these, 106,844 originated from the discovery in the full panel (ALL call). The other 76,286 SNPs were identified using data from only the parents of POP1 (PAR call), which were selected using less stringent filtering. Most SNPs (65.8%) could be identified from the alignment files of both mappers (Additional file 3).

### Mode of inheritance

**Fig. 1:**
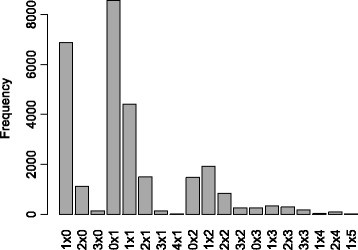
Distribution of marker types in POP1 after the first run of the pipeline (see Methods). Marker types are depicted as “dosage maternal parent” x “dosage paternal parent”. Total number of markers: 28,485

The first run of the SNP dosage scoring pipeline was performed to investigate segregation in POP1. Axiom array signal intensities of all genotyped material were used to estimate allele dosage with a modified version of fitTetra [17] (referred to as fitPoly). In the first run of the pipeline, we estimated dosage of 28,485 markers that were unique and segregated in POP1 (Fig. 1). In the second run of the pipeline, we assumed hexasomic inheritance. The number of scored non-duplicate markers segregating in POP1 was very similar in this second run (Additional file 4). Most of the markers had a dosage of 0 (nulliplex) in one parent and 1 (simplex) in the other. The paternal parent seemed to be more heterozygous compared to the maternal parent considering there are more simplex and duplex markers in the paternal parent than in the maternal parent.

**Fig. 2:**
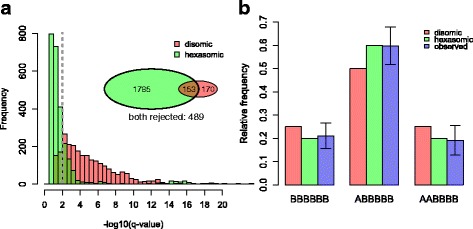
Histogram of q-values of all 2 × 0 markers tested for deviations of disomic and hexasomic segregation using a X^2^ test **a** Inlay: venn diagram of 2 × 0 markers that did not reject disomic inheritance (*red*) and hexasomic inheritance (*green*) at q = 0.01 (*dotted grey line in histogram*). Total number of markers: 2597; markers that were rejected for both types: 489. Barplot of expected genotype frequencies assuming disomic and hexasomic inheritance, and observed average frequency per class **b** Error bars indicate standard deviations

In order to investigate the mode of inheritance in POP1, we tested the segregation of all 2597 2 × 0 markers. None of the 2 × 0 markers showed only simplex scores in the offspring. The markers were subsequently tested for goodness of fit to a 1:2:1 segregation as expected from disomic inheritance, or 1:3:1 segregation as expected from hexasomic inheritance. We used multiple testing corrected *p*-values, q-values, which resulted from a Χ^2^ test of deviations from the two expected segregations. In general, Χ^2^ tests having hexasomic segregation as null hypothesis had higher q-values compared to disomic segregation (Fig. 2a), suggesting better fits to hexasomic segregation. For 1938 out of 2597 SNPs (74.6%) hexasomic inheritance was not rejected at q = 0.01. For 323 SNPs (12.4%) disomic inheritance was not rejected, of which 153 were also not rejected for hexasomic inheretance. For 489 SNPs (18.8%) both segregation types were rejected, indicating skewed segregation or SNP scoring errors. On average, the frequencies in each genotypic class of all 2 × 0 markers, were more similar to hexasomic inheritance than to disomic inheritance (Fig. 2b).

**Fig. 3:**
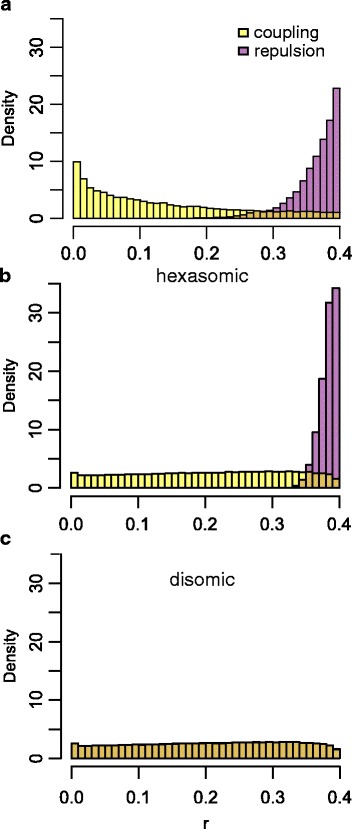
Distribution of recombination frequency (*r*) between simplex x nulliplex markers in repulsion (*purple*) or in coupling phase (*yellow*) in POP1 (**a**) and in a simulated dataset where inheritance was completely hexasomic (**b**) and completely disomic (**c**)

To compare linkages in coupling and repulsion, we calculated the diploid maximum likelihood estimator of *r* between all 1 × 0 markers of POP1 and POP3. For both POP1 and POP3, very large numbers of marker combinations were linked in coupling within a Haldane’s distance of 8 cM (*r* < 0.074), whereas none were linked in repulsion at that distance (Table 1). The minimum *r* from all linkages in repulsion was 0.15 and 0.08 for POP1 and POP3 respectively. We compared the distribution of *r* for repulsion and coupling linkages from POP1 to two simulated datasets (Fig. 3). The simulated datasets differed in preferential pairing; in the first dataset, we imposed full hexasomic inheritance, so random pairing of homologues and in the second full preferential pairing, so disomic inheritance (Fig. 3b and c). In POP1, the distribution of *r* for linkages in repulsion tended towards higher values of *r* which is comparable to the simulated dataset in which we imposed hexasomic inheritance (Fig. 3a).

**Table 1:** Statistics of comparison of repulsion and coupling linkages of markers segregating in POP1 and POP3

			Minimum recombination frequency (*r*)		Linkages within 8 cM^b^ (*r* < 0.074)	
Population	Size^a^	1 × 0 markers	Coupling^c^	Repulsion	Coupling	Repulsion
POP1	398	15,433	0	0.15	606,566	0
POP3	72	6461	0	0.08	46,822	0

^a^After quality filtering of individuals (see methods section)

^b^Haldane’s distance

^c^Estimated phase: coupling or repulsion. Recombination frequency in repulsion was calculated using the diploid maximum likelihood estimator

### Genotyping array validation

We re-ran the SNP dosage scoring pipeline to estimate dosage for all genotyped individuals, which was different from the first run, in which we only aimed to estimate dosage for POP1. For this second run, hexasomic inheritance was assumed and the information on the distinction of the F1 populations and cultivar panel was used. In total, 73,936 markers (40.4%) could be called by fitPoly and had a missing value rate lower than 20%; those SNPs were considered as successfully discovered. Of those, 62,679 segregated as expected assuming hexasomic inheritance from the parental genotypes in at least one of the mapping populations and had a missing value rate lower than 10% (Additional file 5). These markers are suitable for genetic analyses that need high quality marker data, like linkage mapping.

In total 34,068 SNPs were tiled from both 35 base-pair flanking regions and were therefore represented by two independent markers on the genotyping array. These two markers both tag the same SNP. Of these, 17,170 could be scored and segregated as expected in POP1 from at least one of the tiled regions. For 55% (9438) of those SNPs only one of both sides showed clear clustering (Additional file 6). Of the SNPs for which both probes showed clear clustering (7549; 45%), 1.1% (183; 0.6% of total) did not correspond to each other.

**Fig. 4:**
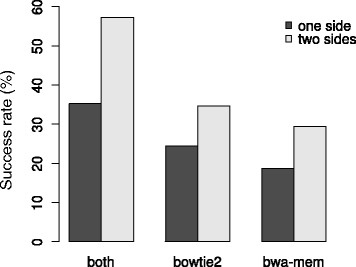
Percentage of segregating SNPs per class in which a SNP was discovered using alignment files of either type of mapping software, or one of the two specifically

Markers that were called from both the bowtie2 and bwa-mem alignment had a higher success rate than markers that were called with either one of the two types of mapping software alone (Fig. 4). Markers called with bowtie2 had a slightly higher success rate than those called with bwa-mem.

**Fig. 5:**
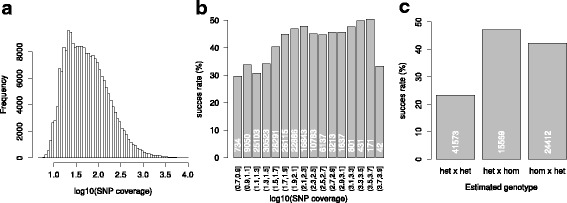
Coverage distribution of selected SNPs, the coverage is represented as the mean coverage of the 13 sequenced genotypes of a SNP locus **a** Success rate (number of SNP loci with a successful dosage call) versus the average SNP coverage in the RNA-seq data per coverage interval, as depicted by the two numbers below each bar **b** Success rate of SNPs from the PAR call versus the estimated genotype (heterozygous: het, or homozygous: hom) of the two parents of POP1 **c** The number of SNP loci per class are written in white numbers inside the bars

The median of the coverage per selected SNP per sequenced genotype was 49 (Fig. 5a). SNPs with a coverage per genotype higher than 100 had a substantially higher success rate compared to SNPs with lower average coverage (Fig. 5b). In the ALL call, we selected only SNPs that were homozygous in at least one genotype. We assumed this would have a positive effect on the success rate. In the PAR call however, also SNPs were allowed that were heterozygous in both parental genotypes assessed. The comparison of the three groups (both heterozygous, only mother heterozygous or only father heterozygous) within the PAR call showed a clear positive effect on the success rate if one of the parents was heterozygous and the other homozygous (Fig. 5c).

### Allelic expression

**Fig. 6:**
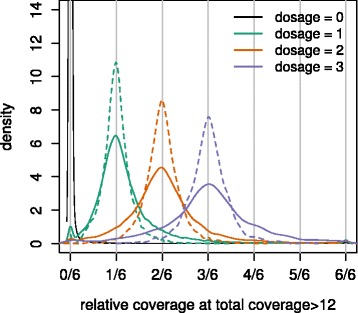
The density of relative coverage of RNA-seq data in each SNP class as genotyped by the Axiom array based on genomic DNA. Total number of analysed SNPs: 52,052. Solid lines represent the observed density and dashed lines represent expected density based on a binomial distribution. The figure represents data that originates from the parents of POP1. SNPs were filtered based on correspondence between parental dosages and observed segregation ratios and had a relative dosage greater than twelve

For the SNPs that segregated as would be expected from the parental dosages in POP1, we compared the genomic dosage of the parents of POP1 with the relative allele coverage in the parents from the RNA-seq data (Fig. 6). The average relative coverage per SNP allele per sequenced genotype matched the expected dosages. Distribution of relative coverage was more dispersed than expected from a binomial distribution, while the difference in dispersion between the binomial distribution and observed distribution was similar over dosages.

## Discussion

### SNP filtering from RNA-seq data

Transcriptome assembly from short-read RNA-seq data of a heterozygous polyploid organism comes with challenges. One of those arises when trying to separate alleles from gene homologues [12]. In sequence data from genomic DNA, unexpected variation in coverage and unexpected numbers of alleles per locus can be used to identify wrongly assembled contigs [42]. However, variation in coverage cannot be used with RNA-seq data, since expression varies strongly between genes. Detection of an unexpected number of alleles is difficult in a hexaploid, as the number of alleles per locus can vary between two and six. In the ALL call, we have therefore tried to select against SNPs that were detected on an assembly of transcripts of two homologous loci, by selecting only SNPs for which at least one genotype was homozygous.

Our selection method seems to have been successful, as SNPs from the PAR call that were heterozygous in both parents had a much lower success rate compared to SNPs from the same call for which one of the two parental genotypes was homozygous. The lower success rate of SNPs that are heterozygous in both parents could also be caused by their higher chance of complex segregation ratios in the progeny. For example, a duplex x duplex (2 × 2) SNP, would have an expected segregation ratio of 1:6:11:6:1 resulting in five clusters for the dosages 0, 1, 2, 3 and 4 respectively. Markers with more than two clusters have a higher chance of missing values or wrongly assigned dosages, as more clusters usually have a higher chance to overlap each other.

It is important to note that we probably invoked a bias towards SNPs with lower dosages. The reasons for this are the selection for SNPs for which there was at least one homozygous individual, and the difficulties with SNP dosage scoring of more complexly segregating SNPs. In line with this expected bias, we found that most of the SNPs we detected were of the 1 × 0 type in the parents of the F1 populations. However, relatively high numbers of 1 × 0 markers are also found in other studies of polysomic polyploids in which there was no such marker selection [1, 23, 29, 36, 43–48]. Moreover, a relatively high frequency of 1 × 0 markers is expected from a population genetics point-of-view. In a breeding germplasm, the dosage of bi-allelic markers tend to become more extreme. This is because the progeny of a parent with a medium-dose SNP (e.g. 3) will segregate in a wide range of dosages, whereas 1 × 0 markers stably segregate in a 1:1 ratio into dosages of 1 and 0. New mutations usually get introduced in a dosage of 1, and have a high chance to remain in the germplasm in low dosages if there is no selection. In conclusion, the observed high frequency of 1 × 0 markers is not only a result of the used SNP selection methods, and the strength of the bias towards lower allele frequencies is therefore difficult to quantify.

The use of two different types of sequence read mapping software resulted in three sets of SNPs: SNPs only identified with either bowtie2 or bwa-mem, and SNPs identified with both mappers. As there is some level of independence between the two SNP calls from both alignments, it would be expected that SNPs discovered with both mappers have a higher success rate [49]. This was indeed the case, SNPs identified with both mappers had a much higher success rate (58% versus <35%).

### Allelic expression

From the segregating SNP markers in our dataset, SNP allele read counts in the transcriptome conformed to their dosage estimated from the array data. Chrysanthemum therefore deviates from the allopolyploids wheat, cotton and camelina that show subgenome-specific expression patterns in a large number of expressed genes [10, 50–52]. In autopolyploids on the other hand, expression patterns are generally conform genomic dosage [53, 54], like we observed in chrysanthemum.

Variation of relative allele coverage in the chrysanthemum read data was larger than would be expected from a binomial distribution. The source of this extra variation could be both biological and technical. The biological reason would be allelic expression imbalance of some loci, which is also common in diploids [55–57]. A technical reason could be allelic bias, caused by higher chance of alignment of reads that exactly match the reference allele compared to the alternative allele or scoring errors, but this should have been visible as deviations from expected relative dosages in our distributions.

### The mode of inheritance

Our dataset gives evidence of complete or near-complete hexasomic inheritance in chrysanthemum. A first indication is the absence of non-segregating 2 × 0 markers. Presence of those type of markers would indicate disomic inheritance. Analysis of the segregation ratios of the 2597 segregating 2 × 0 markers pointed towards hexasomic segregation. Only 6.5% of 2 × 0 markers were rejected for hexasomic segregation and not rejected for disomic segregation. It is likely that a large number of the markers fitting only disomic segregation had genotyping errors or skewed segregation, as the number of markers not fitting any of the two types (18.8%) was much higher.

Conforming to the analysis of 2 × 0 markers, comparison of linkages between 1 × 0 markers in coupling and repulsion phase also pointed towards absence of disomic inheritance in two populations (POP1 and POP3). There were no linkages in repulsion with a distance smaller than 8 cM, while a very large number of marker combinations in coupling were linked within this distance (606,566 and 46,822 for POP1 and POP3 respectively). This is contrary from what would be expected from disomic inheritance, since with disomic inheritance, the ratio between the number of linkages in coupling and in repulsion would be 1:1 [39], irrespective of the threshold of *r*. We did find linkages between markers in repulsion in which *r* was lower than 0.4, the minimum expected *r* in repulsion for full hexasomic inheritance [40]. In the simulated dataset in which we imposed full hexasomic inheritance, we also found linkages with *r* below the threshold. However, there were fewer, and minimum *r* was higher (0.29 compared to 0.15 and 0.08 for POP1 and POP3 respectively). The lower minimum *r* in the real datasets could be caused by incomplete disomic inheritance [40]. However, genotyping errors or commonly occurring lethal allelic combinations leading to segregation distortions [58, 59] are also plausible reasons, and were not taken into account in the simulation.

In a number of studies, the expected distributions of marker dosage in hexaploid F1 populations were used to draw conclusions on the mode of inheritance [1, 29, 36, 47, 48]. These expected distributions are derived from the calculations of da Silva et al. [60]. A polysomic hexaploid would have an expected ratio of 75:25 between simplex and higher-dose markers, whereas this would be 62.5:37.5 for a disomic hexaploid. However, the calculations of da Silva et al. are restricted to populations that originate from a cross between a heterozygous genotype and its derived doubled haploid [60, 61]. The expected frequencies therefore do not apply to F1 populations originating from a cross with other types of parentage, including the populations used in this study and aforementioned studies [1, 29, 36, 47, 48].

Our conclusions deviate from the most recent and elaborate paper on the mode of inheritance in chrysanthemum authored by Klie et al. [1]. We conclude that inheritance in chrysanthemum is completely or nearly completely hexasomic, whereas Klie and colleagues conclude chrysanthemum to be a segmental allopolyploid (in which allopolyploidy is used in the context of mode of inheritance, so disomic). However, the analysis of segregation and repulsion linkage as reported by Klie et al. do not contradict our results. These authors also conclude that their results point to hexasomic inheritance. Despite this, they base their final conclusion on earlier suggestions of disomic inheritance that were wrongly based on observations of prevalence of bivalents during meiosis [32, 62]. As reviewed in the background section, the observation of a predominance of bivalents is not an indication of disomic inheritance, as homologues can still pair with any other homologous chromosomes in a bivalent [34], and prevalence of formation of bivalents seems to be under genetic control in chrysanthemum [35]. Based on earlier work done on the mode of inheritance of chrysanthemum that resulted in conclusive evidence [1, 36, 37], and our results on two biparental populations, we suggest to classify cultivated chrysanthemum as a hexaploid with polysomic inheritance.

## Conclusions

We present an application of the use of next generation sequencing and high-throughput genotyping in hexaploid chrysanthemum. Development of these resources opens up many possibilities for plant improvement at the level of the genome. As a first step, we were able to find conclusive evidence for near-complete hexasomic inheritance on a genome-wide scale. With these resources and the information on the mode of inheritance we will now be able to progress in the development of genomic tools for genetic improvement in chrysanthemum, like linkage mapping and mapping of traits.

## Methods

### Plant materials

A panel of thirteen genotypes was selected for RNA-seq (Additional file 1). We aimed to represent an as broad as possible genetic variation in cut chrysanthemums. Selection was based on flower type, growth habit and absence of less than third degree relationships. The genotypes genotyped with the Axiom array consisted of a biparental population of 405 progeny of which the parents were included in the RNA-seq panel (POP1), three biparental populations consisting of 53 (POP2), 76 (POP3) and 37 (POP4) progeny respectively (Table 2), and a cultivar panel consisting of 63 genotypes. The parents of the biparental populations were not known to have a relationship closer than a third degree.

**Table 2:** Overview of parents used for the biparental populations

	Female parent	Male parent	Size
POP1	DB36451^a^	DB39287^a^	405
POP2	DB41234^a^	DB40360	53
POP3	DB9656^a^	DB9541^a^	76
POP4	DB32141	DB39287^a^	37

^a^Also in the RNA-seq panel

### Sample preparation RNA-seq

Plants were grown according to commercial growing standards in a greenhouse in Maasdijk, the Netherlands. Short photoperiods of 11 h followed longer photoperiods of 14 h to induce flowering. To get coverage for different transcriptomes, and therefore more different transcripts, samples were taken from five different combinations of environments, time points and tissues (Additional file 7). Samples were ground and approximately 100 mg was used for a 1 mL Trizol extraction according to the manufacturers’ protocol (ThermoFisher Scientific, Waltham, MA, USA). After extraction, RNA concentration was estimated using a Nanodrop spectrophotometer (ThermoFisher Scientific, Waltham, MA, USA), and RNA integrity was checked by electrophoresis on a 1.5% agarose gel. Samples were pooled per cultivar in equal ratios. After that, RNA’s were cleaned up using a Qiagen RNeasy column according to manufacturers’ protocol (Qiagen, Venlo, the Netherlands).

### RNA sequencing

Library preparation and sequencing was carried out by GenomeScan B.V. (Leiden, the Netherlands). Library preparation was done using the strand-specific NEBNext Ultra Directional RNA Library Prep kit for Illumina sequencing (New England Biolabs, Ipswich, MA, USA). In short, messenger RNA was isolated from total RNA using oligo-dT magnetic beads. Then, mRNA was fragmented and reverse transcribed into cDNA. The cDNA was ligated to sequencing adapters and amplified using PCR. Fragment size selection was between 400 and 900 basepairs. Clustering and DNA sequencing was performed using the Illumina cBot and HiSeq 2500 (Illumina, San Diego, CA, USA) according to manufacturers’ protocols, resulting in paired-end reads with a length of 126 basepairs at both ends.

### Quality filtering, assembly and mapping

Trimmomatic [63] (v0.33) was used for quality trimming and filtering. Adapter sequences were removed using the ILLUMINACLIP option. Because of lower sequence quality in the leading and trailing ends of the reads, three basepairs were trimmed off at both ends using the LEADING and TRAILING options. Low quality regions were trimmed off using the SLIDINGWINDOW option with a window of four basepairs and a minimum quality of 15. Read pairs were discarded if less than 70 base pairs (bp) remained in one or both reads.

The two parents of POP1 were assembled separately. Trinity [64] version 2.0.6 was used for assembly using a minimum k-mer coverage of 2. Other settings were set at default values. Based on different quality criteria as described in the results section, the assembly of the female parent was selected as reference transcriptome. To reduce contig redundancy, the transcriptome was clustered using uclust [41] at 99% similarity. Samples were mapped to the reference transcriptome using Bowtie2 [65] and bwa-mem [66]. For Bowtie2 (v2.1.0) the --very-sensitive option used and the options −3 and −5 were set to 5 in order to reduce the effect of error-prone read ends on the mapping. For bwa-mem (v 0.7.12), default options were used except for setting the –M option for Picard compatibility. Read duplicates were marked using Picard (http://broadinstitute.github.io/picard/), and duplicates and reads with a map quality smaller than 2 were removed using samtools [67].

### SNP calling and filtering

Our aim was to identify both SNPs that can be used for a wide range of chrysanthemum genotypes and to identify SNPs for linkage mapping in POP1 specifically. To achieve this, we aligned reads of all 13 cultivars (ALL) and reads originating from only the two parents of POP1 (PAR) separately. In addition, we wanted to include SNPs that were called with two different types of mapping software (bowtie2 and bwa-mem). Therefore, four alignment files were created: the reads of the ALL set aligned with bowtie2 and bwa-mem and the reads of the PAR set aligned with bowtie2 and bwa-mem (Additional file 8). SNPs were called using QualitySNP [68] from these four files separately. To reduce the number of false positives and rare SNPs, the option minimalNumberOfReadsPerAlleleP was set to 0.08 for the ALL call and 0.04 for the PAR call. The flanks were set at 35 bp and maxNumberOfSNPsInFlanks was set to 1. A list with marker sequences with 35 bp at each side was exported using the QualitySNP GUI.

We continued with SNP filtering by use of custom made R [69] (v3.1) scripts. All SNPs called from one type of mapping software were combined. From the “variations” output file of QualitySNP, the number of reads for each SNP allele was extracted. SNP-cultivar combinations with a total coverage greater than 12 were used to estimate the zygosity of the cultivars; whether it was homozygous or heterozygous. For each SNP in each genotype the relative coverage of the minor allele was calculated as the fraction of the coverage of the minor allele compared to the total coverage. Genotypes with a relative allele coverage smaller than 0.005 or greater than 0.995 were assigned homozygous and heterozygous otherwise. To select against ambiguous SNP calls, the assigned zygosity (heterozygous or homozygous) were used to filter out groups of SNPs that had the same flanking sequences, originated from different contigs, and showed different zygosities in any of the cultivars.

**Fig. 7:**
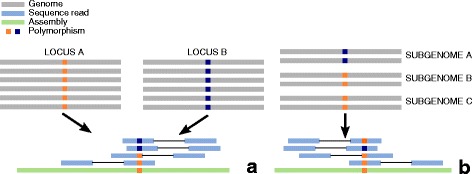
Examples of false positive SNPs against which was selected. In **a** two recently duplicated loci have SNPs between each other but not within. In **b** a SNP that is homozygous and specific for a subgenome. In both situations, all genotypes appear to be heterozygous and SNPs will not segregate in a population

We selected against SNPs that were detected as heterozygous in all genotypes, as they have a large chance of being false positives, or subgenome defining. False positive SNPs can originate from mapping reads originating from two or more recently duplicated genes to one locus, and would appear as a heterozygous SNP in all genotypes (Fig. 7a). Subgenome specific SNPs that are homozygous within the subgenome would also appear heterozygous in all genotypes (Fig. 7b). In both cases, these SNPs are not interesting for linkage mapping, analysis of quantitative trait loci and association studies, as they will not segregate in a population. In order to select against them, only markers were kept that gave at least one heterozygous and one homozygous called genotype in the panel. We did not use this selection for SNPs originating from the alignments of PAR set since SNPs heterozygous in both parents are very informative for linkage mapping, and we did not want to deplete our dataset for these SNP types.

After selection against SNPs heterozygous in all genotypes, flanking sequences including the reference SNP allele were aligned to the reference transcriptome using BLAST with an e-value cut-off of 1e-5. Contigs assembled by Trinity are classified into different hierarchical categories [64]. Markers with a hit in different groups of contigs as separated by the highest hierarchical category (component) or without a 100% hit including the reference allele were discarded. After that, SNPs selected from the two types of alignment software were taken together, and duplicates were removed. Further filtering by the Affymetrix bioinformatics team resulted in a set of 183,130 SNPs of which 34,068 could be tiled from both directions.

### Genotyping with Axiom array

An Axiom genotyping array was designed by Affymetrix. We expected resolution to be an issue for high confidence dosage estimation [18], since for a hexaploid a maximum of seven genotype clusters can be expected instead of the regular three in a diploid and five in a tetraploid. Therefore, each probe was tiled four times instead of the regular two times. Genotyping was performed by Cigene (Ås, Norway). The Affymetrix bioinformatics team postprocessed raw signals of each of the four probe replicates into normalized signal intensities per probe.

### Dosage scoring and quality filtering

Marker dosage was called from ratios of signal intensities of the genotyping array using a modified version of fitTetra [17], that allowed dosage scoring in other ploidy levels than tetraploid. We refer to it as fitPoly. The option p.threshold was set to 0.97. After running fitPoly, a composite quality score was calculated based on conflicts between segregation and parental dosage (allowing for both hexasomic and disomic inheritance), conflicts between scores assigned to replicate samples of the parents, missing parental scores and number of missing values. A threshold for this composite score was determined upon visual inspection of the signal intensities and scored dosages per marker. All markers below this threshold were filtered out. Probes of markers that were tiled from both sides were compared. Probes from one SNP locus with less than 4% different dosage scores were merged into one marker. Others were kept in the dataset as separate markers.

Two individuals of POP1 with more than 5% unexpected dosages based on parental dosages were removed. Then, marker dosages were converted to their most fundamental form, as described by Bourke et al. [44]. After that, markers and individuals with more than 10% missing values were removed. Markers were considered duplicates if all their non-missing dosages were equal. Duplicate markers were grouped and the marker with the least missing values was kept in the dataset as representative. Skewedness of simplex x nulliplex (1 × 0) markers was quantified using the probability of a Χ^2^ test assuming a 1:1 segregation. Probabilities were corrected for multiple testing (referred to as q-values) and markers with q < 0.01 were removed. After filtering, the dataset consisted of two parents and 398 F1 progeny genotyped with 28,485 markers.

After analysis of segregation of markers in POP1, it became clear that preferential pairing was absent. We re-ran the pipeline without filtering for duplicate markers on all biparental populations assuming hexasomic inheritance. In this case we also supplied fitPoly with information on the population each sample belonged to (four F1 populations, their parents, or the cultivar panel), which allowed fitPoly to use the expected segregation ratios to improve model fitting. To investigate repulsion linkage in POP3, and to investigate the marker distribution of POP1 compared to the first run, we repeated the post fitPoly processing of markers to those populations as described for the first run.

### Pedigree simulation

In order to estimate the expected distribution of repulsion linkages with known modes of inheritance, we simulated F1 populations of 400 individuals each. We used PedigreeSim V2.0 [70] with 900 simplex x nulliplex (1 × 0) markers randomly placed on each of the 9 chromosomes. All chromosomes had a length of 100 cM, the centromeres were positioned at 50 cM. Hexasomic and disomic inheritance were simulated by setting the prefPairing parameter at 0 and 1 respectively for each chromosome. For each of these situations one F1 population was simulated.

### Linkage analysis and statistics

Recombination frequency (*r*) and logarithm of odds were calculated as described in Van Ooijen and Jansen [71]. Statistical analysis, other calculations and plotting were performed with R [69]. Venn diagrams were plotted with the R package VennDiagram [72]. Multiple testing correction was performed with the R package qvalue [73].

## Additional files

Additional file 1: Overview of genotypes used for RNA-seq. (PDF 8 kb)

Additional file 2: Statistics of assemblies of the two parents. (PDF 8 kb)

Additional file 3: Venn diagram of the number of SNP markers called with bowtie2 and bwa-mem. (PDF 59 kb)

Additional file 4: Distribution of marker types in POP1 after the second run of the pipeline (see Methods). Marker types are depicted as “dosage maternal parent” x “dosage paternal parent”. Total number of markers: 27,902. (PDF 19 kb)

Additional file 5: Venn diagram of number of markers segregating in different biparental populations. (PDF 126 kb)

Additional file 6: Pie chart of comparisons of both SNP assays of markers tiled from both sides segregating in POP1. Corresponding: both assays gave less than 4% conflicting dosages. One missing: one of the assays could not be called by fitPoly. Not corresponding: two assays gave different results. Total number of SNPs: 17,170. (PDF 9 kb)

Additional file 7: Environment and tissue type of different samples. (PDF 6 kb)

Additional file 8: Overview of SNP calling steps. For more information, see materials and methods. QSNP: QualitySNP. (PDF 22 kb)

## Abbreviations

ALL: SNP marker discovery pipeline in the full panel of 13 genotypes
bp: Base pairs
PAR: SNP marker discovery pipeline in the parents of POP1
POP: Population
QSNP: QualitySNP
*r*: Recombination frequency
RNA-seq: RNA Sequencing
SNP: Single Nucleotide Polymorphism

## References

[1] Klie M, Schie S, Linde M, Debener T (2014). The type of ploidy of chrysanthemum is not black or white: a comparison of a molecular approach to published cytological methods. Front Plant Sci.

[2] Elshire RJ, Glaubitz JC, Sun Q, Poland JA, Kawamoto K, Buckler ES (2011). A robust, simple genotyping-by-sequencing (GBS) approach for high diversity species. PLoS One.

[3] Baird NA, Etter PD, Atwood TS, Currey MC, Shiver AL, Lewis ZA (2008). Rapid SNP discovery and genetic mapping using sequenced RAD markers. PLoS One.

[4] Saintenac C, Jiang D, Akhunov ED (2011). Targeted analysis of nucleotide and copy number variation by exon capture in allotetraploid wheat genome. Genome Biol.

[5] Uitdewilligen JGAML, Wolters AMA, D’hoop BB, Borm TJA, Visser RGF, van Eck HJ (2013). A next-generation sequencing method for genotyping-by-sequencing of highly heterozygous Autotetraploid potato. PLoS One.

[6] Koning-Boucoiran CFS, Esselink GD, Vukosavljev M, van’t Westende WPC, Gitonga VW, Krens FA, et al. Using RNA-Seq to assemble a rose transcriptome with more than 13,000 full-length expressed genes and to develop the WagRhSNP 68k axiom SNP array for rose (Rosa L.) Front Plant Sci. 2015; 6:249. 10.3389/fpls.2015.00249 PMC4404716 25954285

[7] Wang S, Wong D, Forrest K, Allen A, Chao S, Huang BE (2014). Characterization of polyploid wheat genomic diversity using a high-density 90 000 single nucleotide polymorphism array. Plant Biotechnol J.

[8] Choi K, Henderson IR (2015). Meiotic recombination hotspots - a comparative view. Plant J.

[9] Chen ZJ (2007). Genetic and epigenetic mechanisms for gene expression and phenotypic variation in plant Polyploids. Annu Rev Plant Biol.

[10] Leach LJ, Belfield EJ, Jiang C, Brown C, Mithani A, Harberd NP (2014). Patterns of homoeologous gene expression shown by RNA sequencing in hexaploid bread wheat. BMC Genomics.

[11] Osborn TC, Chris Pires J, Birchler JA, Auger DL, Chen ZJ, Lee HS (2003). Understanding mechanisms of novel gene expression in polyploids. Trends Genet.

[12] Shahin A, van Gurp T, Peters SA, Visser RG, van Tuyl JM, Arens P (2012). SNP markers retrieval for a non-model species: a practical approach. BMC Res Notes.

[13] Ganal MW, Durstewitz G, Polley A, Bérard A, Buckler ES, Charcosset A (2011). A large maize (Zea Mays L.) SNP genotyping array: development and germplasm genotyping, and genetic mapping to compare with the B73 reference genome. PLoS One.

[14] Chagné D, Crowhurst RN, Troggio M, Davey MW, Gilmore B, Lawley C (2012). Genome-wide SNP detection, validation, and development of an 8K SNP array for apple. PLoS One.

[15] Bassil NV, Davis TM, Zhang H, Ficklin S, Mittmann M, Webster T (2015). Development and preliminary evaluation of a 90 K axiom® SNP array for the allo-octoploid cultivated strawberry Fragaria × ananassa. BMC Genomics.

[16] Vos PG, Uitdewilligen JGAML, Voorrips RE, Visser RGF, van Eck HJ (2015). Development and analysis of a 20K SNP array for potato (Solanum Tuberosum): an insight into the breeding history. Theor Appl Genet Springer Berlin Heidelberg.

[17] Voorrips RE, Gort G, Vosman B (2011). Genotype calling in tetraploid species from bi-allelic marker data using mixture models. BMC Bioinformatics..

[18] Grandke F, Singh P, Heuven HCM, de Haan JR, Metzler D (2016). Advantages of continuous genotype values over genotype classes for GWAS in higher polyploids: a comparative study in hexaploid chrysanthemum. BMC Genomics.

[19] Ramsey J, Schemske DW (1998). Pathways, mechanisms, and rates of Polyploid formation in flowering plants. Annu Rev Ecol Syst.

[20] Doyle JJ, Sherman-Broyles S. Double trouble: taxonomy and definitions of polyploidy. New Phytol. 2016. 10.1111/nph.14276 28000935

[21] Ramsey J, Schemske DW (2002). Neopolyploidy in flowering plants. Annu Rev Ecol Syst.

[22] Zhang J, Esselink GD, Che D, Fougère-Danezan M, Arens P, Smulders MJM (2013). The diploid origins of allopolyploid rose species studied using single nucleotide polymorphism haplotypes flanking a microsatellite repeat. J Hortic Sci Biotechnol.

[23] Vukosavljev M, Arens P, Voorrips RE, van’t Westende WP, Esselink G, Bourke PM, et al. High-density SNP-based genetic maps for the parents of an outcrossed and a selfed tetraploid garden rose cross, inferred from admixed progeny using the 68k rose SNP array. Hortic Res. 2016; 3:16052. 10.1038/hortres.2016.52 PMC5080978 27818777

[24] Koning-Boucoiran CFS, Gitonga VW, Yan Z, Dolstra O, van der Linden CG, van der Schoot J (2012). The mode of inheritance in tetraploid cut roses. Theor Appl Genet.

[25] Allendorf FW, Danzmann RG (1997). Secondary tetrasomic segregation of MDH-B and preferential pairing of homeologues in rainbow trout. Genetics.

[26] Nguepjop JR, Tossim H-A, Bell JM, Rami J-F, Sharma S, Courtois B (2016). Evidence of genomic exchanges between Homeologous chromosomes in a cross of peanut with newly Synthetized Allotetraploid hybrids. Front Plant Sci.

[27] Leal-Bertioli S, Shirasawa K, Abernathy B, Moretzsohn M, Chavarro C, Clevenger J (2015). Tetrasomic recombination is surprisingly frequent in allotetraploid Arachis. Genetics.

[28] Stift M, Berenos C, Kuperus P, van Tienderen PH (2008). Segregation models for disomic, tetrasomic and intermediate inheritance in tetraploids: a general procedure applied to Rorippa (yellow cress) microsatellite data. Genetics.

[29] De Backer M. Characterization and detection of Puccinia horiana on chrysanthemum for resistance breeding and sustainable control. Ghent: Ghent University; 2012.

[30] Anderson NO. Chrysanthemum. In: Anderson NO, editor. Flower Breeding and Genetics: Issues, Challenges and Opportunities for the 21st Century. Dordrecht, The Netherlands: Springer; 2007. p. 389–437.

[31] Dai S-L, Wang W-K, Li M-X, Xu Y-X (2005). Phylogenetic relationship of Dendranthema (DC.) des Moul. Revealed by fluorescent in situ hybridization. J Integr Plant Biol.

[32] Chen F Di, Li FT, Chen SM, Guan ZY, Fang WM. Meiosis and pollen germinability in small-flowered anemone type chrysanthemum cultivars. Plant Syst Evol 2009; 280:143–151.

[33] Roxas NJL, Tashiro Y, Miyazaki S, Isshiki S, Takeshita A (1995). Meiosis and pollen fertility in Higo chrysanthemum (Dendranthema x grandiflorum (Ramat.) Kitam.). J Japanese Soc Hortic Sci.

[34] Stebbins GL (1940). The significance of polyploidy in plant evolution. Am Nat.

[35] Watanabe K (1983). Studies on the control of diploid-like meiosis in polyploid taxa of chrysanthemum. Theor Appl Genet.

[36] Park SK, Arens P, Esselink D, Lim JH, Shin HK (2015). Analysis of inheritance mode in chrysanthemum using EST-derived SSR markers. Sci Hortic.

[37] Langton FA (1989). Inheritance in Chrysanthemum Morifolium Ramat. Heredity.

[38] De Jong J, Rademaker W (1986). The reaction of chrysanthemum cultivars to Puccinia horiana and the inheritance of resistance. Euphytica.

[39] Wu K, Burnquist W, Sorrells M, Tew T (1992). The detection and estimation of linkage in polyploids using single-dose restriction fragments. Theor Appl Genet.

[40] Qu L, Hancock JF (2001). Detecting and mapping repulsion-phase linkage in polyploids with polysomic inheritance. Theor Appl Genet.

[41] Edgar RC (2010). Search and clustering orders of magnitude faster than BLAST. Bioinformatics.

[42] McKinney GJ, Waples RK, Seeb LW, Seeb JE. Paralogs are revealed by proportion of heterozygotes and deviations in read ratios in genotyping by sequencing data from natural populations. Mol Ecol Resour. 2016;17:656–69. 10.1111/1755-0998.12613 27762098

[43] Bourke PM, Arens P, Voorrips RE, Esselink GD, Koning-Boucoiran CFS, van ‘t Westende WPC, et al. Partial preferential chromosome pairing is genotype dependent in tetraploid rose. Plant J 2017; 90:330–343. 10.1111/tpj.13496 28142191

[44] Bourke PM, Voorrips RE, Kranenburg T, Jansen J, Visser RG, Maliepaard C. Integrating haplotype-specific linkage maps in autotetraploid potato using SNP markers. Theor Appl Genet. 2016:1–36. 10.1007/s00122-016-2768-1 PMC5069339 27561740

[45] Hackett CA, McLean K, Bryan GJ. Linkage analysis and QTL mapping using SNP dosage data in a Tetraploid potato mapping population. Nelson JC, editor. PLoS One. 2013; 8:e63939. 10.1371/journal.pone.0063939 PMC3660524 23704960

[46] Cervantes-Flores JC, Yencho GC, Kriegner A, Pecota KV, Faulk MA, Mwanga ROM (2008). Development of a genetic linkage map and identification of homologous linkage groups in sweetpotato using multiple-dose AFLP markers. Mol Breed.

[47] Kriegner A, Cervantes J, Burg K, Mwanga R, Zhang D (2003). A genetic linkage map of sweetpotato [Ipomoea Batatas (L.) lam.] based on AFLP markers. Mol Breed.

[48] Ukoskit K, Thompson P (1997). Autopolyploidy versus allopolyploidy and low-density randomly amplified polymorphic DNA linkage maps of sweetpotato. J Am Soc Hortic Sci.

[49] Clevenger J, Chavarro C, Pearl SA, Ozias-Akins P, Jackson SA (2015). Single nucleotide polymorphism identification in Polyploids: a review, example, and recommendations. Mol Plant.

[50] Adams KL, Cronn R, Percifield R, Wendel JF (2003). Genes duplicated by polyploidy show unequal contributions to the transcriptome and organ-specific reciprocal silencing. Proc Natl Acad Sci U S A.

[51] Zhang T, Hu Y, Jiang W, Fang L, Guan X, Chen J (2015). Sequencing of allotetraploid cotton (Gossypium Hirsutum L. acc. TM-1) provides a resource for fiber improvement. Nat Biotechnol.

[52] Kagale S, Koh C, Nixon J, Bollina V, Clarke WE, Tuteja R (2014). The emerging biofuel crop Camelina Sativa retains a highly undifferentiated hexaploid genome structure. Nat Commun.

[53] Albertin W, Brabant P, Catrice O, Eber F, Jenczewski E, Chèvre AM (2005). Autopolyploidy in cabbage (Brassica Oleracea L.) does not alter significantly the proteomes of green tissues. Proteomics.

[54] Church SA, Spaulding EJ (2009). Gene expression in a wild autopolyploid sunflower series. J Hered.

[55] Vogelstein B, Kinzler KW, Yan H, Yuan W, Velculescu VE (2002). Allelic variation in human gene expression. Science.

[56] Heap GA, Yang JHM, Downes K, Healy BC, Hunt KA, Bockett N (2009). Genome-wide analysis of allelic expression imbalance in human primary cells by high-throughput transcriptome resequencing. Hum Mol Genet.

[57] Bell GDM, Kane NC, Rieseberg LH, Adams KL (2013). RNA-seq analysis of allele-specific expression, hybrid effects, and regulatory divergence in hybrids compared with their parents from natural populations. Genome Biol Evol.

[58] Manrique-Carpintero NC, Coombs JJ, Veilleux RE, Buell CR, Douches DS (2016). Comparative analysis of regions with distorted segregation in three diploid populations of potato. G3 Genes|Genomes|Genetics.

[59] Liu X, Guo L, You J, Liu X, He Y, Yuan J (2010). Progress of segregation distortion in genetic mapping of plants. Res J Agron.

[60] da Silva JAG, Sorrells ME, Burnquist WL, Tanksley SD (1993). RFLP linkage map and genome analysis of Saccharum Spontaneum. Genome.

[61] Al-janabi SM, Honeycutt RJ, Mcclelland M, Sobral BWS (1993). A genetic linkage map of Saccharum Spontaneum L. “SES 208”. Genetics.

[62] Li C, Chen S, Chen F, Li J, Fang W (2011). Cytogenetic study of three edible chrysanthemum cultivars. Russ J Genet.

[63] Bolger AM, Lohse M, Usadel B (2014). Trimmomatic: a flexible trimmer for Illumina sequence data. Bioinformatics.

[64] Haas BJ, Papanicolaou A, Yassour M, Grabherr M, Blood PD, Bowden J (2013). De novo transcript sequence reconstruction from RNA-seq using the trinity platform for reference generation and analysis. Nat Protoc.

[65] Langmead B, Salzberg SL (2012). Fast gapped-read alignment with bowtie 2. Nat Methods.

[66] Li H, Durbin R (2010). Fast and accurate long-read alignment with burrows-wheeler transform. Bioinformatics.

[67] Li H, Handsaker B, Wysoker A, Fennell T, Ruan J, Homer N (2009). The sequence alignment/map format and SAMtools. Bioinformatics.

[68] Nijveen H, van Kaauwen M, Esselink DG, Hoegen B, Vosman B (2013). QualitySNPng: a user-friendly SNP detection and visualization tool. Nucleic Acids Res.

[69] R Core Team. R: a language and environment for statistical computing. Vienna, Austria: R Foundation for Statistical Computing; 2014.

[70] Voorrips RE, Maliepaard C (2012). The simulation of meiosis in diploid and tetraploid organisms using various genetic models. BMC Bioinformatics.

[71] Van Ooijen JW, Jansen J (2013). Genetic mapping in experimental populations.

[72] Chen H, Boutros PC. VennDiagram: a package for the generation of highly-customizable Venn and Euler diagrams in R. BMC Bioinformatics. 2011;12:35. 10.1186/1471-2105-12-35 PMC3041657 21269502

[73] Dabney A, Storey JD. qvalue: q-value estimation for false discovery rate control. 2014. R package version 1.38.0.

