# Supplementary material for: Conclusive evidence for hexasomic inheritance in chrysanthemum based on analysis of a 183 k SNP array
Source: BMC Genomics. 2017 Aug 7;18:585. doi: 10.1186/s12864-017-4003-0 (PMC5547472; doi:10.1186/s12864-017-4003-0)
Supplement: Supplementary file 1 — Overview of genotypes used for RNA-seq. (PDF 8 kb) [file 12864_2017_4003_MOESM1_ESM.pdf]

genotype	flower type	flower colour
DB36451	single	white
DB39287	single	white
DB9541	single	red
DB9656	small-flowered	white
DB10957	single	red
DB13124	single	purple
DB13601	double	white
DB21697	double	white
DB28990	semi-double	purple
DB30600	double	white
DB34154	single	white
DB41234	double	green
