# Supplementary material for: Conclusive evidence for hexasomic inheritance in chrysanthemum based on analysis of a 183 k SNP array
Source: BMC Genomics. 2017 Aug 7;18:585. doi: 10.1186/s12864-017-4003-0 (PMC5547472; doi:10.1186/s12864-017-4003-0)
Supplement: Supplementary file 2 — Statistics of assemblies of the two parents. (PDF 8 kb) [file 12864_2017_4003_MOESM2_ESM.pdf]

sample	Number of reads	uclust	Number of contigs	average contig length (bp)	n50 (bp)	n10 (bp)	longest 1000 contig length (bp)
**DB36451**	105660926	n	270186	628.10	841	2354	4513.33
		y	227213	654.06	867	2236	4130.06
**DB39287**	103582212	n	275397	618.84	811	2317	4556.42
		y	231634	644.52	839	2191	4137.46
