# Supplementary figures and images for: Conclusive evidence for hexasomic inheritance in chrysanthemum based on analysis of a 183 k SNP array
Source: BMC Genomics. 2017 Aug 7;18:585. doi: 10.1186/s12864-017-4003-0 (PMC5547472; doi:10.1186/s12864-017-4003-0)

bowtie2

bwa-mem

33117

120523

29490

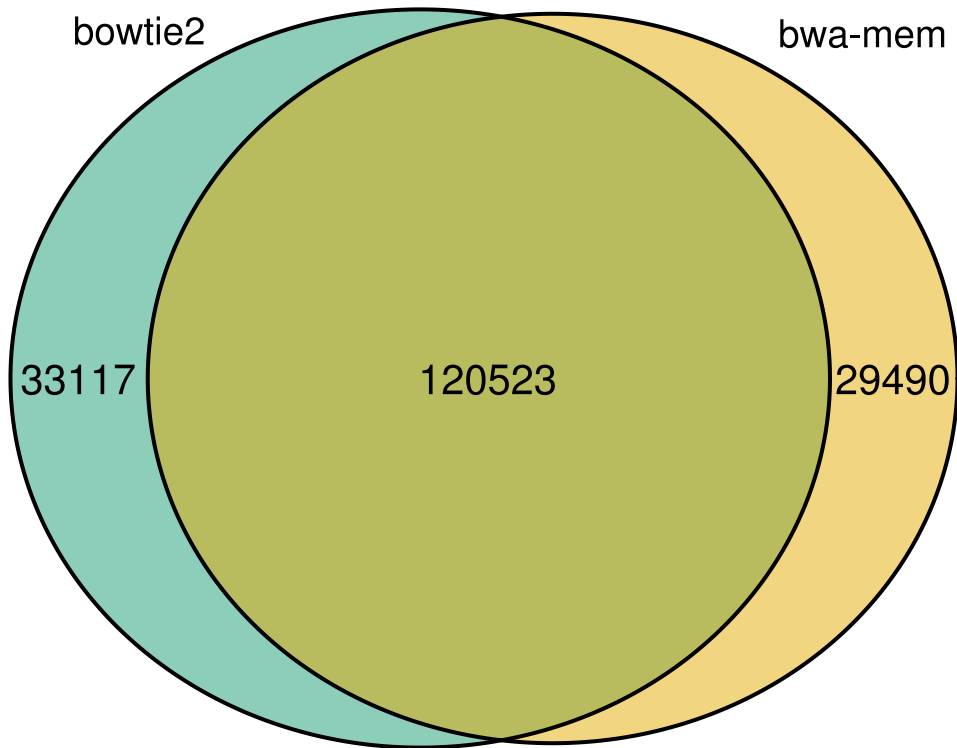

Supplement: Supplementary file 3 — Venn diagram of the number of SNP markers called with bowtie2 and bwa-mem. (PDF 59 kb) [file 12864_2017_4003_MOESM3_ESM.pdf]

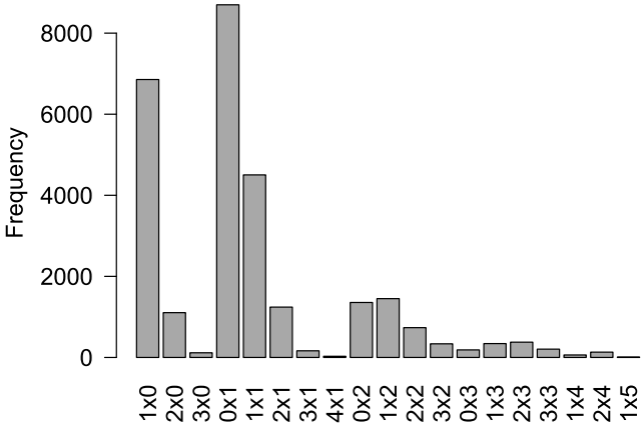

Supplement: Supplementary file 4 — Distribution of marker types in POP1 after the second run of the pipeline (see Methods). Marker types are depicted as “dosage maternal parent” x “dosage paternal parent”. Total number of markers: 27,902. (PDF 19 kb) [file 12864_2017_4003_MOESM4_ESM.pdf]

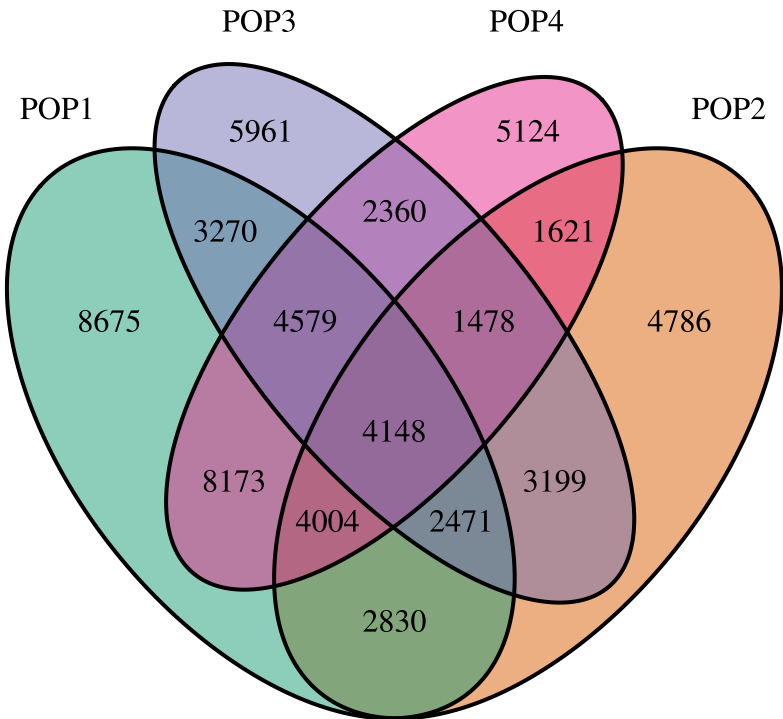

Supplement: Supplementary file 5 — Venn diagram of number of markers segregating in different biparental populations. (PDF 126 kb) [file 12864_2017_4003_MOESM5_ESM.pdf]

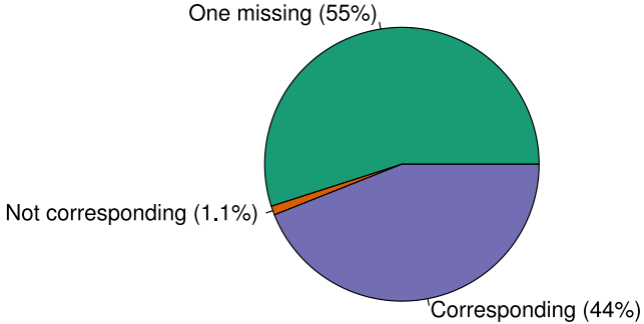

Supplement: Supplementary file 6 — Pie chart of comparisons of both SNP assays of markers tiled from both sides segregating in POP1. Corresponding: both assays gave less than 4% conflicting dosages. One missing: one of the assays could not be called by fitPoly. Not corresponding: two assays gave different results. Total number of SNPs: 17,170. (PDF 9 kb) [file 12864_2017_4003_MOESM6_ESM.pdf]

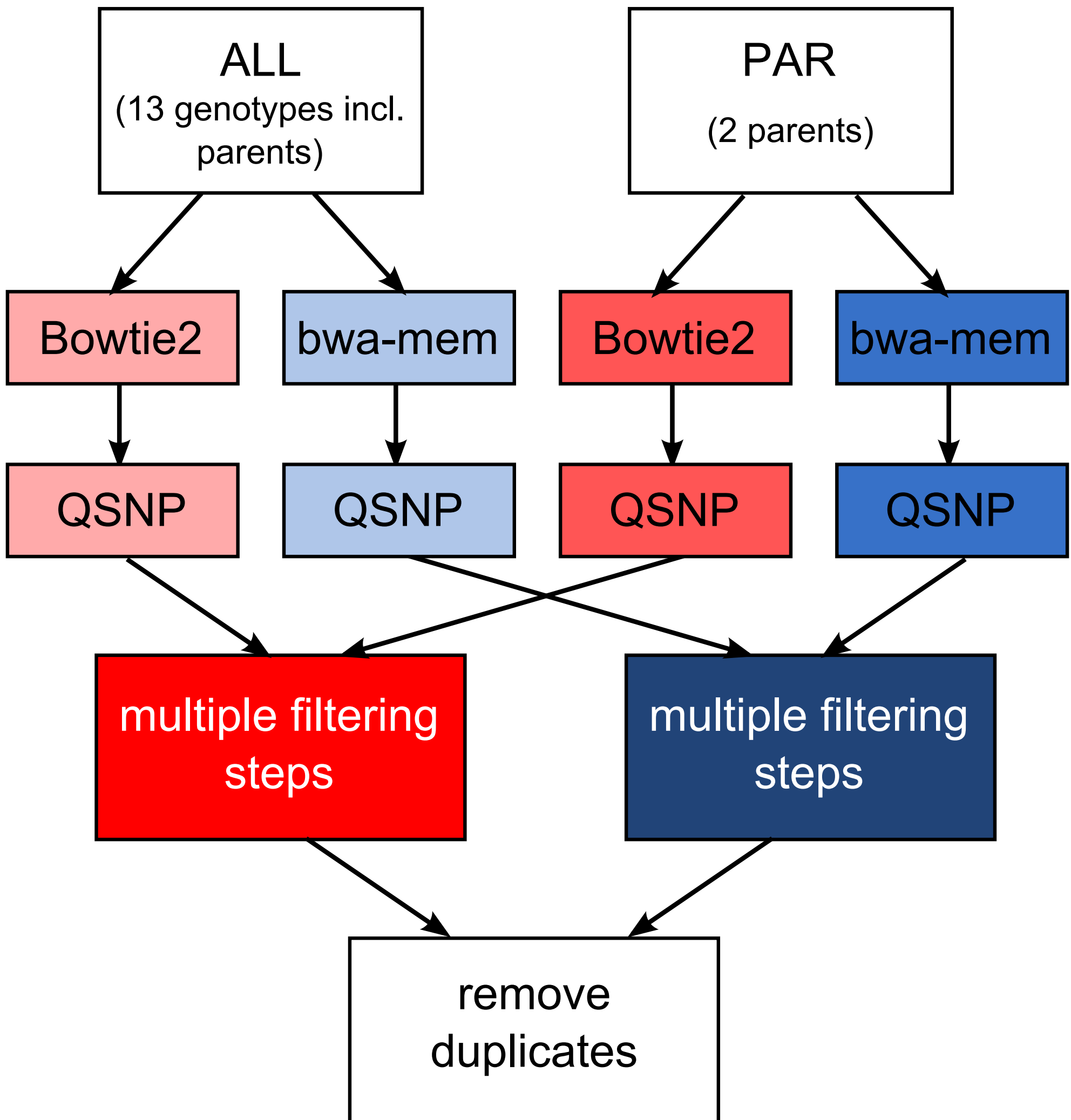

Supplement: Supplementary file 8 — Overview of SNP calling steps. For more information, see materials and methods. QSNP: QualitySNP. (PDF 22 kb) [file 12864_2017_4003_MOESM8_ESM.pdf]
