# Supplementary material for: Conclusive evidence for hexasomic inheritance in chrysanthemum based on analysis of a 183 k SNP array
Source: BMC Genomics. 2017 Aug 7;18:585. doi: 10.1186/s12864-017-4003-0 (PMC5547472; doi:10.1186/s12864-017-4003-0)
Supplement: Supplementary file 7 — Environment and tissue type of different samples. (PDF 6 kb) [file 12864_2017_4003_MOESM7_ESM.pdf]

sample	type of tissue	days at 14h photoperiod	days at 11 hour photoperiod
1	Vegetative shoots	10	0
2	Vegetative shoots	12	3
3	Green flower buds	13	29
4	Green flower buds	12	36
5	Colouring flower buds	13	52
